# Factors that lead to changes in sexual behaviours after a negative HIV test: protocol for a prospective cohort study in Kinshasa

**DOI:** 10.1186/s12889-016-3285-5

**Published:** 2016-07-20

**Authors:** Silvia Carlos, Francis Nzakimuena, Gabriel Reina, Cristina Lopez-del Burgo, Eduardo Burgueño, Adolphe Ndarabu, Alfonso Osorio, Jokin de Irala

**Affiliations:** University of Navarra, Department of Preventive Medicine and Public Health, C/Irunlarrea, 1. 31080 Pamplona, Spain; IdiSNA, Navarra Institute for Health Research, Pamplona, Spain; University of Navarra, Institute for Culture and Society (ICS), Education of Affectivity and Human Sexuality, Pamplona, Spain; CECFOR/Monkole Hospital, Kinshasa, Democratic Republic of Congo; University of Kinshasa, School of Public Health, Kinshasa, Democratic Republic of Congo; Clinic University of Navarra, Clinical Microbiology Department, Pamplona, Spain; University of Navarra, School of Education and Psychology, Pamplona, Spain

**Keywords:** HIV, Voluntary Counseling and Testing (VCT), Negative test, Sexual, Behaviour, Knowledge, Cohort, Africa

## Abstract

**Background:**

Considering the high percentage of couples in which one or both members are HIV negative, the frequency of transmission among non-regular partners and the probabilities of non-disclosure, attention should be paid to people getting a negative HIV test at the Voluntary Counseling and Testing (VCT). Research has shown that a negative HIV test may be followed by a change in sexual behaviours. In Sub-Saharan Africa, where most HIV infections occur, there are few studies that have analysed the factors associated with changes in sexual risk behaviours after a negative HIV test at the VCT clinic. The aim of this project is to evaluate the specific factors associated with changes in sexual behaviours, three months after a negative result in an HIV test, and to analyse the effect of counseling and testing on HIV-related knowledge of participants in an outpatient centre of Kinshasa (Democratic Republic of Congo).

**Methods and design:**

Prospective cohort study from December 2014 until March 2016. People 15–60 year old that received VCT at Monkole Hospital (Kinshasa) were followed three months after they got a negative HIV test. In a face-to-face interview, participants replied to a baseline and a follow-up research questionnaire on HIV-related knowledge, attitudes and behaviours. At follow-up respondents were also offered a new HIV test and additional HIV counseling. Four hundred and fifteen participants completed the baseline questionnaire and 363 (87 %) came back for their 3-month follow up.

**Discussion:**

This is the first longitudinal study in the DRC that evaluates the factors associated with changes in sexual behaviours after a negative HIV test at the VCT. Participants attending the VCT services within a clinical setting are a good study population as they can be good transmitters of preventive information for other people with no access to health facilities.

## Background

HIV Voluntary Counseling and Testing (VCT) is a critical strategy for HIV prevention. In addition to an HIV screening test, VCT includes a pre-test and a post-test counseling session. The pre-test counseling (which can be individual, partner- or group-based) should comprise a personal risk evaluation, the explanation of any misconceptions related to HIV/AIDS and the promotion of preventive measures to avoid and reduce HIV infection and transmission. Post-test counseling, for those participants that get a positive result, should include explanations on how to communicate their result, how to avoid new transmissions and where to get clinical and social services for the infection control. For those with a negative HIV test, post-test counseling should include an explanation about the infection window period and also the preventive strategies to assure they remain seronegative and adherent to the effective measures recommended for HIV prevention [1–3]. Considering the high percentage of couples in which one or both members are HIV negative [4–8], the HIV sexual transmission risks [9, 10], the frequency of transmission among non-regular partners [11] and the probabilities of non-disclosure [4], post-test counseling for those uninfected is really critical to prevent new HIV cases, which currently arise mostly as a result of early onset of sex, multiple serial and concurrent partnerships and incorrect and inconsistent condom use.

It has been shown that in some cases people increase their sexual risk behaviours after receiving a negative test. In Sub-Saharan Africa some longitudinal studies have detected almost a doubling in sexual risk behaviours after a negative test [12, 13], an increase in multiple lifetime and concurrent partners [14, 15], a higher sex frequency [16], an increase in sex with a non-regular partner for those HIV negative in serodiscordant couples [17, 18] or a low risk perception for those whose partners tested negative [19–21]. Regarding condom use, results are not consistent. Some authors have reported that condom use may increase [17] but others have not found a significant change [22–35]. The literature also suggests that VCT only affects people’s behavior when the test results are a surprise [36–39] but there is still few information regarding the specific factors associated with a change in sexual risk [40, 41].

Within Sub-Saharan Africa, Uganda was the first country offering VCT services in 1990 [8] and later in 1998, the national Demographic and Health Surveys (DHS) and AIDS Indicator Surveys (AIS) started to collect information about HIV testing from an increasing number of Sub-Saharan countries: data on whether respondents have ever been tested, the time of the most recent test or about the knowledge of the results. These surveys do not usually register the number of times people get tested, except from the 2011 Uganda AIS, in which most participants reported to have been tested once or twice [42]. To date, there are data available from nearly 50 African surveys and although a general increase in testing is shown, the use of the VCT services varies among the different countries and the different sociodemographic conditions, with estimated proportion of people ever HIV tested increasing from 1.1 % among women in Chad in 2004 up to 80.2 % among women in Zambia in 2014 [5, 43].

In the Democratic Republic of Congo (DRC) (country with a generalized HIV epidemic and an estimated 1.2 % HIV prevalence in the general population), only 15 % of adult men and 20 % adult women from the general population have been tested for HIV, and 6 % of males and 9 % of females among the 15–24 year-olds. Kinshasa, the capital city (with an estimated 1.6 % HIV prevalence in the general population, 2.6 % for women and 0.3 % for men), has the highest frequencies of HIV testing around the country with 34 % of men, 49 % of women and 55 % of pregnant women having been screened and having obtained their results [44, 45]. In all cases, as shown for many other African settings, in the DRC people that get HIV tested are mainly women and adults and belong mostly to the highest economical and educational level (from 2 % up to 22 % from the lowest to the highest wealth quintile and from 2 % among people with no education to 14 % in those with secondary education, respectively) [42, 46].

In the DRC few studies have evaluated the effects of VCT on sexual behaviours. Early in the epidemic in 1991, when VCT services were still not available, Kamenga and collaborators [47] carried out a prospective study that evaluated the effect of work-based VCT in serodiscordant couples, finding an increase in extramarital sex among men with a negative test. Two years later another prospective study evaluated the impact of VCT on contraception and sexual behaviours among child-bearing women, finding that HIV negative women reported a lower contraceptive use than previously planned [48]. In 2000, still previous to antiretroviral use in the DRC (first introduced in 2005 [49]), Ryder et al*.* analysed the effect of VCT on HIV and pregnancy incidence among serodiscordant married workers. They found, again, that when seronegative men knew their wife was HIV positive, they increased the frequency of extramarital sex (mainly protected, after condoms were provided by the study personnel) [17]. The last study in the DRC with data related to VCT took place in 2011 and it was focused on studying the attitudes towards HIV screening among pregnant women and their couples. Most men in the study wrongly believed that their wife’s HIV test result should be the same as their own serostatus and consequently, they didn’t get HIV tested [21].

VCT can be carried out at the community level (through mobile units, at workplace, schools, churches or even home-based) or within clinical settings, being client-initiated or provider-initiated [46, 50, 51]. Although it is not always easy for the population in Sub-Saharan Africa to have access to health-care facilities and usually community-based VCT reaches people earlier in the course of the infection [46], the healthcare context is optimal: it is an entry point for HIV/AIDS care for those testing positive and also it is a critical opportunity to link the VCT attendees to other vulnerable family or community members with no access to health facilities.

Therefore, we designed this study considering that no previous longitudinal studies in the DRC had evaluated the factors associated with a change in sexual risk behaviours, after getting a negative HIV result, among people from the general population receiving VCT within a clinical context. Since 2010 the research team has been working with VCT attendees in the same clinical setting and consequently most of the methodology and personnel are well established and details on this team can be found elsewere [52].

This project aims to evaluate: (1) the prevalence of sexual risk behaviours 3 months after getting a negative HIV test at the VCT session in a health-care centre in Kinshasa; (2) the association between sexual risk levels with baseline factors, such as socio-ecological determinants, personal HIV risk perception, previous HIV testing, reasons for attending VCT, knowledge of HIV and its prevention, information about HIV received, sexual behaviours and STI symptoms and diagnosis; (3) the effect of the information received at the baseline pre- and post-test VCT sessions, on the HIV risk perception, knowledge and sexual risk taking at follow-up; and (4) the HIV preventive needs of the population that have a negative HIV test at VCT. This study aims to be a scientific contribution for the evaluation of new strategies for the future at the post-test counseling sessions in order to prevent new HIV infections, as well as other sexually transmitted infections (STIs), specifically on those receiving HIV negative tests.

## Methods

### Study design

Prospective cohort study from December 2014 to March 2016 at Monkole Hospital, in Kinshasa (DRC). All enrolled participants having a negative HIV test at the VCT clinic were followed up for a 3-month period.

### Study setting

Monkole Hospital, located in South-Western Kinshasa, is a private health care center that is integrated within the Congolese public health system with regards to HIV prevention and care and other health issues. It was built in 1991 and since 2005 Monkole is getting support from the Global Fund for HIV education, for the sensibilization and treatment of users and has been the reference hospital of 5 out of 35 Health Zones in Kinshasa with regards to HIV care and treatment. Monkole Hospital now covers a total population of around 300,000 inhabitants within Mont Ngafula I Health Zone regarding HIV.

The population receiving medical care at Monkole Hospital is representative of the general population in Kinshasa and mainly includes women and young adults, with an average secondary education, working on agriculture and family businesses and having a low socoeconomic status [44, 53].

Monkole Hospital has an outpatient center where people can receive VCT (both client- and provider-initiated) as well as HIV/AIDS care. The patients testing HIV positive at the VCT cite are usually followed up and begin the antiretroviral treatment if they have CD4 levels below 500/mm^3^. Considering the last available data, around 1000 patients are followed up at Monkole and are on antiretroviral treatment. Based on the national guidelines the antiretroviral drugs currently available at Monkole are zidovudine, lamivudine, didanosine, nevirapine, efavirenz, tenofovir, abacavir and lopinavir/ritonavir. For the HIV/AIDS support, Monkole is also receiving funding from Elisabeth Glaser Paediatric Aids Foundation (EGPAF).

### Participants

**Fig. 1:**
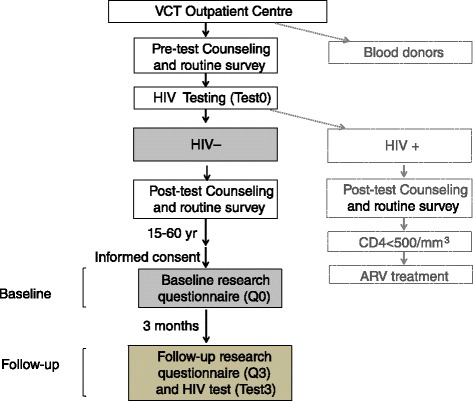
Project flow chart

People aged 15–60 years old that attended the VCT centre at Monkole Hospital and got a negative HIV test result were included in this study (Fig. 1). The last Demographic Health Survey from the DRC included data from 15–59 year-old men and from women aged 15–49. We wanted to collect information for a wider age range among both men and women. Firstly, young people aged 15–24 represent around 40 % of the new HIV infections among people 15 years old and older. This is the reason why we included participants not only from late adolescence but also from middle adolescence (15–17 year-old) [54, 55]. In addition, it has been estimated that nearly 15 % of HIV-infected adults are 50 years old or older and that the numbers will nearly triple in Sub-Saharan Africa in the near future, with more than 1 in 4 HIV-infected patients being from that age group by 2040 [56–59]. They are also at risk of HIV, based on their sexual behaviours but additionally they exhibit biological factors that also facilitate the infection, such as the presence of a thinner vaginal epithelium, decreased mucus production or immune changes in postmenopausal women that facilitate HIV infection [60]. Moreover, people in this age group usually have a low perception of their own risk of acquiring HIV [56].

People with an HIV positive test were excluded from the study as well as blood donors attending the same outpatient centre (Fig. 1). In the study we did not have pregnant women because they attend specific prenatal care centers.

### Sample size

Annually around 500 people 15–60 year-old attend Monkole hospital for VCT and around 90 % get a negative HIV test. Considering a 1-year period and having had a 99 % response rate in our previous case-control study in Monkole [52] we estimated to be able to include around 445 HIV negative VCT participants at baseline. Considering a minimum 70 % retention rate at 3 months [47, 61–65], we estimated that we could have around 310 HIV negative participants with a 3 month-follow-up.

From December 2014 to March 2016, 415 VCT participants with an HIV negative test completed the baseline data and 363 were followed up (87 %).

The dependent variables of this study are changes in behaviours (sexual initiation, multiple sexual partnerships, condom use) and HIV knowledge. Taking into account the sample size estimation criteria that about 10 events are needed for each parameter included in a regression multivariate model, we are therefore confident to have sufficient events in all our analyses to account for confounding in our study [66–68].

### Data collection

#### The questionnaires

The study is based on face-to-face interview-administered questionnaires. All people that accepted to participate in the study were offered to respond to two research questionnaires (baseline and follow-up), in addition to the two brief routine pre-test and post-test surveys that were part of the counselling sessions and that gathered sociodemographic and behavioural information. Following the National Guidelines, when participants were re-tested (at the 3-month follow-up) the pre- and post-test routine surveys were again carried out.

Both baseline and follow-up research questionnaires include mainly closed questions. The baseline questionnaire (48 questions) collects data regarding sociodemograhic factors (sex, age, birth and growing up place, orphanity, educational level, educational setting (public/private; rural/urban), exposure to mass media, socioeconomic status, religion and religiosity), health-related aspects (health perception, alcohol and drug use and STI symptoms and diagnosis), previous HIV testing and HIV risk perception, HIV-related knowledge, attitudes and sexual behaviours as well as on the HIV information sources, frequency and messages received.

The follow-up questionnaire is similar but shorter (26 questions), and additionally includes some questions regarding the VCT service 3 months prior to follow up but without requesting sociodemographic information again.

Both questionnaires are based on previously validated surveys, including the HIV-Knowledge-27-Scale, specifically suited to Sub-Saharan populations [69].

**Fig. 2:**
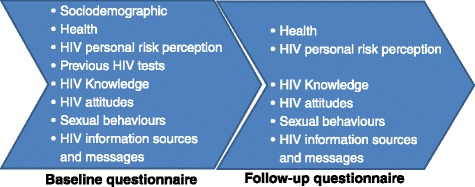
Main sections of the baseline and 3-month follow-up questionnaires

The study questionnaires were first written in Spanish, translated to Congolese French and then backtranslated into Spanish to verify that the first translation was correct. It took an average of 35 and 20 min to complete the baseline and follow-up questonnaires, respectively (Fig. 2).

### Interviewers

For both the baseline and the follow-up questionnaires two nurses (male and female) were available to carry out the interviews. They were not part of the routine VCT team at Monkole hospital so that they could focus on the specific project tasks, but they were familiar with the overall performance of VCT and with the related sensitive aspects. Before the beginning of the study they all received a specific training for the study implementation and a local researcher supervised their work daily.

Additionally, before the beginning of the project, a pilot study was carried out with a subgroup of 18 participants in order to make sure that all procedures worked correctly.

### HIV tests

Since 1985, UNAIDS and WHO have always recommended rapid HIV diagnosis tests, so that results can be communicated as soon as possible and the post-test counseling can be carried out straightforwardly after having a negative or positive test. The test should always fulfill the ‘3 Cs’: confidential, counseled and consented [70].

At Monkole Hospital, blood samples are analysed for HIV with the rapid test HIV Determine® VIH-1/2 (Abbott). A positive result is confirmed with other two different tests: DoubleCheckGold® (Orgenics) and Unigold® (Trinity Biotech). The HIV test result is usually communicated within 15–30 min.

When an undetermined result is found (a positive first-line test and at least one negative second-line test), the HIV diagnosis is repeated two weeks after. If again the HIV test result is undetermined, a confirmatory analysis is recommended. However, due to limited economical resources, it needs to be done in an external laboratory. Patients with an initial undetermined HIV test result were also included in this study.

In the present study, when participants with a negative HIV test came back again for the 3-month follow-up they were re-tested for HIV [71].

### Follow-up

For all the respondents with a negative HIV test at baseline, an appointment was set to go back to Monkole afer a 3-month period for the follow-up. One week before the follow-up visit they received a reminding call.

In order to assure a high retention rate, participants received a transportation fee when they arrived at the hospital for the follow-up.

Eighty-seven percent of participants scheduled for a follow-up visit returned to Monkole for their appointment.

### Data registration

To ensure the best data quality, all information from the routine VCT pre-test and post-test surveys of Monkole Hospital, as well as from the research baseline and follow-up questionnaires, have been double-checked both at Monkole Hospital and at the University of Navarra (all questionnaires were scanned and sent to the University of Navarra for that purpose).

### Planned statistical analysis

All the statistical analysis will be performed using Stata 12.1 program.

Our study sample consists of patients yielding a negative HIV test and counseling sessions and that were followed-up to evaluate changes in HIV-related behavioural and knowledge variables. The “exposure variables” in this study will therefore consist of specific combinations of individual and community-level factors that will be collected at the baseline questionnares and used as predictors of change in outcomes at follow-up, using multivariate statistics [72]. The following baseline variables will be analysed as possible predictors of HIV risk behavior/knowledge change at follow-up and will therefore be considered in the multivariate regression models as possible predictors or confounding variables to be adjusted for: sociodemographic variables (i.e., sex, age, education, economic level, marital status, religion), participant’s personal HIV risk perception, reason for attending the VCT, previous HIV testing, knowledge of HIV and its prevention, information about HIV received, sexual behaviours and STI symptoms and diagnosis.

In our project we are interested in studying both, increases and decreases of risk from baseline to follow-up. Non-conditional logistic regressions will be carried out, with ‘increased and decreased risk’ as dichotomous dependent variables. Variables named ‘increased risk’ and ‘decreased risk’will be calculated to evaluate the changes in sexual behaviours and HIV knowledge after a 3-month follow-up (Table 1). The following sexual risk behaviours will be analyzed: 1) ‘adolescent sexual initiation’, 2) ‘concurrent sexual partners’, 3) ‘multiple lifetime sexual partners’, 4) ‘partner with multiple sexual partners’, 5) ‘inconsistent condom use’, 6) ‘anal sex’ and 7) ‘forced sex’. All the previous dichotomous variables will be subtracted (follow-up – baseline) and thus yielding new variables (change from baseline to follow-up) with values 0 (no change), +1 (increase) and -1 (decrease). These new variables will then be recategorized to build our dependent outcomes. Outcome variables measuring increased risk would be coded as 0 (if change variable is 0 or -1) and 1 (if change variable is 1). Outcome variables measuring decreased risk would be coded 0 (if change variable is 0 or +1) and 1 (if change variable es -1). Also, the ‘changes in knowledge’ will be similarly calculated (Table 2) considering the following dichotomous items as correct answers: ‘someone HIV+ can look healthy’, ‘AIDS is not caused by witchcraft or God’s punishment’, ‘the HIV is not transmitted through mosquito bite’, ‘the HIV is not transmitted through social kissing’, ‘HIV can be transmitted in one single unprotected sexual relationship’, ‘HIV can be transmitted in a protected relationship with an infected person’, ‘HIV can be transmitted when having 3–4 sexual lifetime partners regardless of condom use’ and ‘HIV can be transmitted when having concurrent partners regardless of condom use’. ‘Don’t know’ answers will be considered equivalent to not yielding the right answer.

**Table 1:** Sexual behaviour both at baseline and follow-up

Sexual Risk behaviour	Baseline questionnaire (Q0)	Follow-up questionnaire (Q3)
1) Adolescent sexual initiation	‘Have you ever had sex?’ 0 = no; 1 = yes ‘Do you have any sexual partner?’ Number of partners, recategorized as 0 = none; 1 = ≥1partner	‘During the last 3 months, did you have sex?’ 0 = no; 1 = yes ‘Do you have any sexual partner?’ Number of partners, recategorized as 0 = none; 1 = ≥1partner
2) Concurrent sexual partners	‘Do you have any sexual partner?’ Number of partners, recategorized as 0/1 = no concurrent; 1 = ≥2partners ‘During the last 6 months what is the maximum number of concurrent partners you had?’ Number of partners, recategorized as 0/1 = no concurrent; 1 = ≥2partners	‘Do you have any sexual partner?’ Number of partners, recategorized as 0/1 = no concurrent; 1 = ≥2partners ‘During the last 3 months what is the maximum number of concurrent partners you had?’ Number of partners, recategorized as 0/1 = no concurrent; 1 = ≥2partners
3) Multiple lifetime sexual partners	‘During the last 6 months, how many sexual partners have you had?’ Number recategorized as 0/1 = no; 1 = ≥2partners	‘During the last 3 months, how many sexual partners did you have?’ Number recategorized as 0/1 = no; 1 = ≥2partner
4) Partner with multiple sexual partners	‘During the last 6 months, how many sexual partners did your partner have?’ 1; 2–5; >5 recategorized as 0 = no multiple; 1 = ≥2partners	‘During the last 3 months, how many sexual partners did your partner have?’ 1; 2–5; >5 recategorized as 0 = no multiple; 1 = ≥2partners
5) Inconsistent condom use	‘Have you ever used a condom?’ 0 = never; 1 = sometimes; 2 = almost always; 3 = always recategorized as 0 = no consistent; 1 = inconsistent	‘In the last 3 months, did you used a condom?’ 0 = never; 1 = sometimes; 2 = almost always; 3 = always recategorized as 0 = no consistent; 1 = inconsistent
6) Anal sex	‘Have you ever had anal sex?’ 0 = never; 1 = sometimes; 2 = frequently recategorized as 0 = no; 1 = yes	‘In the last 3 months, did you have anal sex?’ 0 = never; 1 = sometimes; 2 = frequently recategorized as 0 = no; 1 = yes
7) Forced sex	‘Have you ever had forced sex?’ 0 = no; 1 = yes	‘In the last 3 months, did you have forced sex?’ 0 = no; 1 = yes

**Table 2:** HIV knowledge variables assessed both at baseline and follow-up

HIV-related knowledge	Baseline questionnaire (Q0)	Follow-up questionnaire (Q3)
Knowledge about HIV infection and transmission	‘Someone HIV+ can look healthy’ 0: incorrect = no/ I don’t know; 1: correct = yes	‘Someone HIV+ can look healthy’ 0: incorrect = no/ I don’t know; 1: correct = yes
	‘AIDS is caused by witchcraft or God’s punishment’ 0: incorrect = yes/I don’t know; 1: correct = no	‘AIDS is caused by witchcraft or God’s punishment’ 0: incorrect = yes/I don’t know; 1: correct = no
	‘HIV is transmitted through social kissing’ 0: incorrect = yes/I don’t know; 1: correct = no	‘HIV is transmitted through social kissing’ 0: incorrect = yes/I don’t know; 1: correct = no
	‘HIV is transmitted through mosquito bite’ 0: incorrect = yes/I don’t know; 1: correct = no	‘HIV is transmitted through mosquito bite’ 0: incorrect = yes/I don’t know; 1: correct = no
	‘HIV can be transmitted in 1 single unprotected sexual relationship’ 0: incorrect = no/I don’t know; 1: correct = yes	‘HIV can be transmitted in one single unprotected sexual relationship’ 0: incorrect = no/I don’t know; 1: correct = yes
	‘HIV can be transmitted in a protected relationship with an infected person’ 0: incorrect = no/ I don’t know; 1: correct = yes	‘HIV can be transmitted in a protected relationship with an infected person’ 0 = no; 1 = yes; 2 = I don’t know
	‘HIV can be transmitted when having 3-4 sexual lifetime partners regardless of condom use’ 0: incorrect = no/ I don’t know; 1: correct = yes	‘HIV can be transmitted when having 3-4 sexual lifetime partners regardless of condom use’ 0: incorrect = no/ I don’t know; 1: correct = yes
	‘HIV can be transmitted when having concurrent partners regardless of condom use’ 0: incorrect = no/ I don’t know; 1: correct = yes	‘HIV can be transmitted when having concurrent partners regardless of condom use’ 0: incorrect = no/ I don’t know; 1: correct = yes

We will additionally evaluate what effects did the VCT session have on the participants by analysing their personal perceptions on the program: ‘perceived change in the personal HIV risk’, ‘perceived change in sexual behaviours’, ‘perceived change in HIV knowledge’ and ‘perceived change in STIs knowledge’ (no change/low change/enough change/high change).

### Ethical issues

The study was approved by the Ethical Research Committees of the University of Kinshasa and of the University of Navarra.

Participation was completely voluntary and all the individuals that accepted to participate in the study gave their written informed consent to respond to the study questionnaires and to get an additional follow-up HIV test done (Fig. 1). For participants that were minors, their legal guardians needed to sign the informed consent.

Both the baseline and follow-up questionnaires were anonymized and they included an identification code that allowed researchers to match baseline and follow-up data. With regards to confidentiality, the interviews took place in private rooms and both the interviewers and the data-entry staff signed a form declaring they would keep standards of confidentiality. The registration forms that included both the names and the identification codes of the participants were kept in a safe locked place.

## Discussion

Findings from this study are expected to be useful to evaluate the specific information people testing HIV negative should receive at VCT provided in healthcare centers in Kinshasa.

### Limitations and strengths

This project has the following limitations. Firstly, all collected data are based on self-report and could therefore be subject to the phenomenon of social desirability bias and consequently under-reporting of specific risk behaviours. However, the selection of skilled interviewers and the efforts made to develop and pilot the questionnaires has hopefully reduced the likelyhood or at least the magnitude of this bias. As well, same-sex interviewers and private rooms probably also contributed to make this bias less likely [73, 74]. Secondly, participants in this study knew the HIV test result when they responded to the baseline questionnaire, as it was one of our selection criteria, but we believe that most people are less likely to conceal the truth about their behaviors and knowledge after being informed about having a negative status, once the stress of the waiting time has passed [36]. In any case, before the data analysis, we will check the quality of the information by analysing any possible inconsistencies. Finally, we are analysing changes in the short-term (a 3-month follow-up period) and thus will not be able to preditc long term changes, but this decision was made to assure a lower attrition rate.

Despite these limitations, this study has several strengths. Firstly, this is the first longitudinal study in the DRC that evaluates the factors associated with changes in sexual behaviours and HIV knowledge after a negative HIV test at a VCT cite. Secondly, the working group and methodology is quite consolidated after a project already previously carried out at Monkole Hospital with 1630 participants attending the VCT clinic [52]. Thirdly, this project collects data from a wider age range (15–60 year-olds), including late adulthood and thus collecting information from a wider age group of sexually active persons at risk of HIV infection. Finally, as mentioned before, participants attending the VCT services within a clinical setting are a good study population as they can be good transmitters of preventive information for other people with no access to health facilities.

## Abbreviations

AIS, AIDS Indicator Surveys; DHS, demographic health survey; DRC, Democratic Republic of Congo; STI, sexually transmitted infection; VCT, Voluntary Counseling and Testing
